# Hidden Depths: Unveiling Endometriosis in the Canal of Nuck

**DOI:** 10.7759/cureus.64975

**Published:** 2024-07-20

**Authors:** Devyansh Nimodia, Pratapsingh Parihar, Pankaj Banode, Sakshi S Dudhe, Prasad Desale, Shubhi Gaur

**Affiliations:** 1 Radiodiagnosis, Jawaharlal Nehru Medical College, Datta Meghe Institute of Higher Education and Research, Wardha, IND; 2 Interventional Radiology, Jawaharlal Nehru Medical College, Datta Meghe Institute of Higher Education and Research, Wardha, IND

**Keywords:** extrauterine endometriosis, radiological findings, pelvic pain, gynecological anomaly, canal of nuck, endometriosis

## Abstract

Endometriosis located within the canal of Nuck represents a highly uncommon occurrence, often posing a diagnostic challenge due to its atypical site and varied clinical presentations. The case of a 31-year-old female who presented with groin swelling and subsequent suprapubic pain for a duration of two years is described in this study. Utilizing magnetic resonance imaging (MRI), a cystic lesion was detected within the canal of Nuck, raising suspicion of endometriosis. Surgical exploration confirmed the presence of endometrial implants, supporting the initial radiological findings. This particular case emphasizes the significance of imaging techniques in diagnosing endometriosis in unusual sites, thereby enabling timely interventions and enhancing patient outcomes. Furthermore, it underscores the necessity of a multidisciplinary approach involving radiologists, gynecologists, and surgeons in ensuring comprehensive care for such patients. In the subsequent sections, we endeavor to present a unique instance of endometriosis within the canal of Nuck, a condition scarcely documented in the existing global literature. Our objective is to heighten awareness and encourage the consideration of endometriosis as a potential differential diagnosis in females presenting with inguinal masses and pelvic discomfort.

## Introduction

The occurrence of endometriosis in the canal of Nuck is a rare medical condition initially described by Cullen in 1896 [1]. Endometriosis, a prevalent gynecological ailment affecting 5%-10% of women during their reproductive years [1], is characterized by the ectopic implantation of functional endometrial tissue outside the uterus, commonly within the ovaries and peritoneum. However, instances of endometriosis in extrapelvic locations, with an estimated incidence of 0.8% [2], predominantly involve extraperitoneal structures such as the hernia sac and the integument. Various alternative sites include the vagina, vulva, cervix, perineum, inguinal canal, urinary system, gastrointestinal tract, pulmonary structures, extremities, integument, and central nervous system [2]. While the peritoneum and pelvic organs are well-established sites of endometriosis, contributing to chronic pelvic pain, dysmenorrhea, and infertility, it is noteworthy that remote locations like the pleura can also be affected, potentially leading to catamenial pneumothorax [3]. Embryologically, the parietal peritoneum extends into the inguinal canal and the labium majus alongside the round ligament of the uterus in females; nevertheless, it typically undergoes complete obliteration within the initial year of life. Persistence of this structure results in the formation of the canal of Nuck, named after Anton Nuck, a Dutch anatomist who documented the initial case in 1691. The canal of Nuck may harbor various entities such as hydrocele, hernia, and cyst [4]; however, endometriosis is an infrequent occurrence in this region and may manifest as a nonspecific soft tissue mass in the groin, prompting consultations with plastic or general surgeons. Given that gynecology services traditionally manage endometriosis, this case underscores the necessity for general surgeons to understand this condition and its distinction from other groin soft tissue masses prior to surgical intervention.

## Case presentation

**Figure 1:**
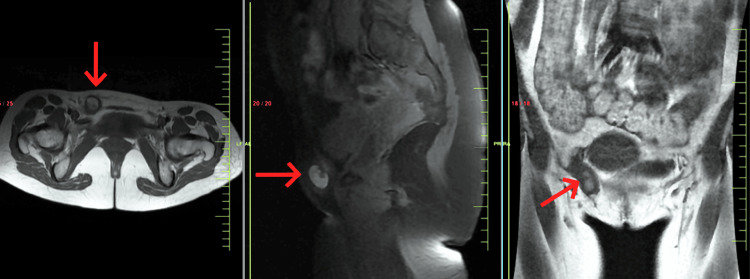
Pelvic MRI showing a well-defined round mass approximately 2.4 x 2.4 x 2.2 cm in size extending from the right inguinal canal into the subcutaneous plane of the right lateral pubic area appearing as hypointense with central hyperintensity on axial and coronal section FSE T1-weighted, sagittal section FSE fatSAT T1-weighted images MRI: Magnetic resonance imaging; FSE: fast spin-echo; FSE fatSAT T1-weighted images: fast spin-echo fat-suppressed T1-weighted images

**Figure 2:**
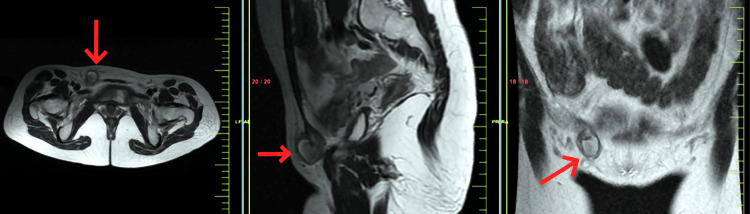
Pelvic MRI showing a well-defined round mass approximately 2.4 x 2.4 x 2.2 cm in size extending from the right inguinal canal into the subcutaneous plane of the right lateral pubic area appearing as hypointense with central hyperintensity on axial, coronal, and sagittal section frFSE T2-weighted images MRI: Magnetic resonance imaging; frFSE T2-weighted images: fast recovery fast spin-echo T2-weighted images

**Figure 3:**
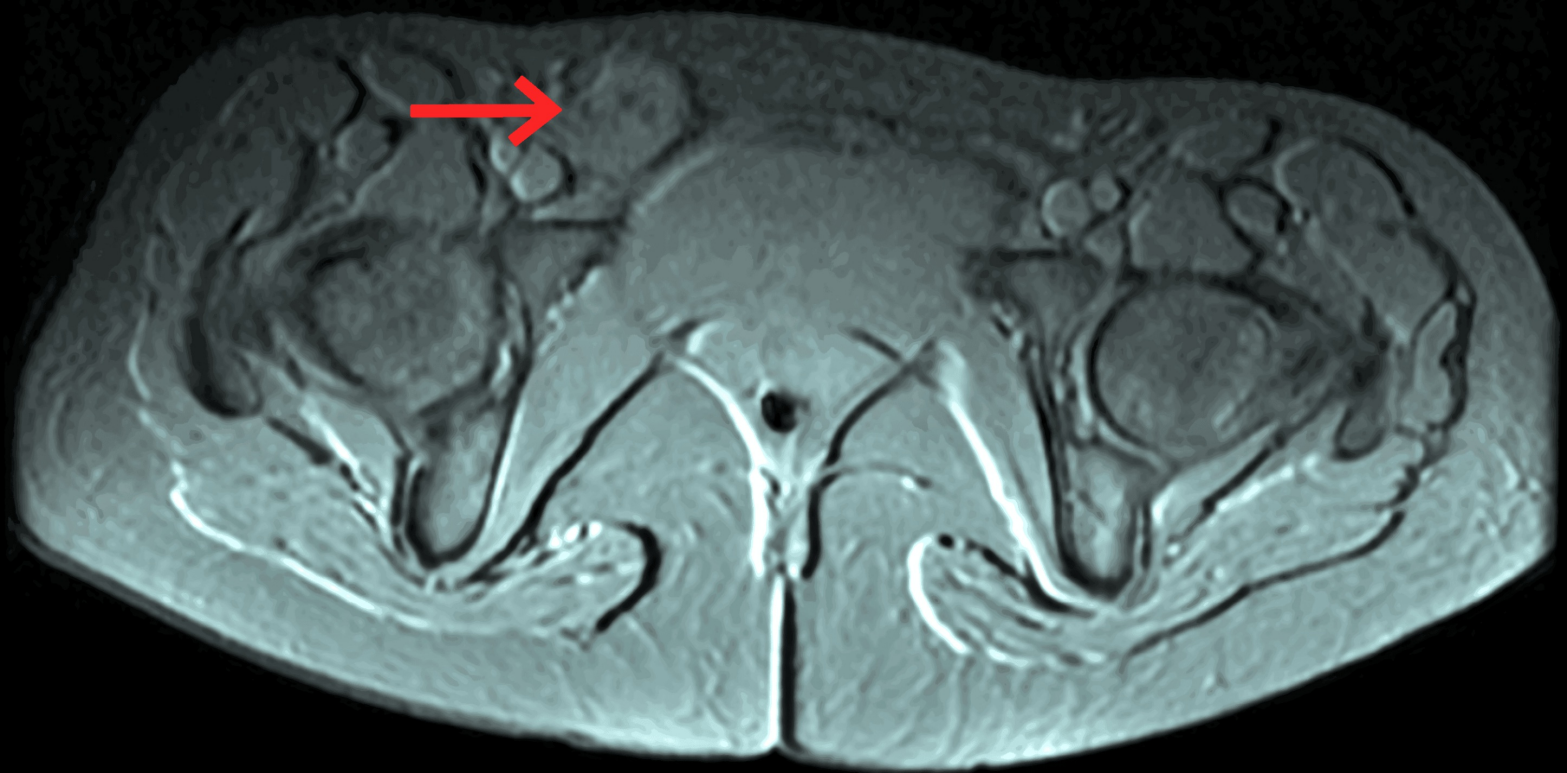
Pelvic MRI showing a well-defined round mass approximately 2.4 x 2.4 x 2.2 cm in size extending from the right inguinal canal into the subcutaneous plane of the right lateral pubic area revealing few areas of blooming on the T2* GRE sequence MRI: Magnetic resonance imaging; T2* GRE sequence: T2-star gradient echo sequence

A 31-year-old healthy nulliparous, married female presented to the surgical clinic with a swelling in her right groin, accompanied by mild discomfort. The patient had no history of infertility. The patient's symptoms progressed over time, and she noticed the presence of a mass approximately nine months prior to seeking medical attention. She experienced an initial episode of dull aching pain while running, which was severe enough to necessitate cessation of physical activity. At times, she detected a small reducible lump in her right suprapubic region. She reported a history of irregular menstrual cycles with cyclical pain during menstruation, correlating with changes in the size of the mass. Upon physical examination, a 2.5 cm subcutaneous thickening was identified to the right of the midline overlying the patient's right pubis, eliciting pain upon palpation. No concomitant pelvic disorders were observed, and normal bowel habits were reported. There was no record of trauma, and the patient's medical history was unremarkable, with the absence of associated symptoms. The patient reported a history of catamenial pain at the time of evaluation and had no history of urinary tract infections. On laboratory investigations, the patient had elevated levels of cancer antigen 125 (CA 125) of 55 U/mL. Fine-needle aspiration (FNA) was done which did not give any definitive diagnosis. Subsequently, a pelvic magnetic resonance imaging (MRI) was ordered and showed a well-defined round mass with an approximate size of 2.4 x 2.4 x 2.2 cm extending from the right inguinal canal into the subcutaneous plane of the right lateral pubic area that was hypointense with central hyperintensity on axial and coronal section fast spin-echo (FSE) T1-weighted, sagittal section FSE fat-suppressed (fatSAT) T1-weighted images in Figure 1 and axial, coronal, and sagittal section fast recovery FSE (frFSE) T2-weighted images in Figure 2. Few areas of blooming were noted on the gradient echo (GRE) sequence in Figure 3. Imaging characteristics indicated a potential diagnosis of inflammatory disease. Considering the clinical presentation, anatomical location of the mass and the MRI findings, the presence of endometriosis within the canal of Nuck was suspected. Definitive management involved a laparoscopic excision of the mass, followed by a conclusive excisional biopsy of the lesion. Surgical laparoscopic exploration unveiled an approximately 2.4 x 2.2 cm, brown-colored mass enclosed in a saclike structure, closely adjacent to the round ligament. Pathological analysis of the specimen confirmed the presence of endometriosis, aligning with both surgical and histological observations. Patient was advised follow-up after six months. Patient reported after six months with no signs and symptoms.

## Discussion

Endometriosis is characterized by the ectopic implantation of functional endometrial tissue outside the uterine cavity, commonly involving the ovaries and peritoneum. A rare occurrence known as endometriosis in the canal of Nuck was initially delineated by Cullen in 1896 [1]. The canal of Nuck represents a vestigial structure originating from the processus vaginalis peritonei, manifesting as a fingerlike protrusion of the parietal peritoneum alongside the round ligament, extending through the inguinal canal into the labium majus. Normally, this canal undergoes spontaneous obliteration within the initial year of life. However, in certain instances, persistence of the patent canal of Nuck facilitates a conduit between the peritoneal cavity and the inguinal canal, potentially serving as a locus for the implantation of endometrial tissue. This persistence of the canal of Nuck lends support to the hypothesis of retrograde transport or migration of endometrial cells into the extraperitoneal domain by establishing a connection between the peritoneal cavity and the inguinal canal [5]. Endometriosis affecting the canal of Nuck manifests as a nonspecific soft tissue mass in the groin area, accompanied by cyclic pain experienced during menstruation. This phenomenon is attributed to the lesion's responsiveness to hormonal fluctuations at the onset of menstruation, leading to an inflammatory reaction. Additionally, it is theorized that extrapelvic endometriosis may exhibit a lack of hormonal receptors, resulting in potential presentation without cyclic symptomatology [6].

The chronic pelvic pain, as defined by the American College of Obstetricians and Gynecologists, is characterized as noncyclic pain enduring for a duration of six months or more. Typically, this form of pain localizes to specific areas such as the anatomic pelvis, anterior abdominal wall below or at the umbilicus, the lumbosacral back, or the buttocks, and is of a magnitude adequate to induce functional disability or necessitate medical intervention. A variety of common etiologies contribute to chronic pelvic pain in females, encompassing pelvic organ pathologies like bladder conditions and fibromyalgia [7].

The etiology of endometriosis remains a subject of debate within the scientific community; however, the prevailing hypothesis suggests that the dissemination of endometrial tissue is a result of retrograde menstruation. While dysmenorrhea, pelvic pain, and infertility are the most commonly observed symptoms, they lack specificity, and atypical presentations are frequent in cases where endometriotic implants are located in unconventional sites. In instances where endometriosis impacts the canal of Nuck, the primary clinical manifestation tends to be the presence of a groin inguinal mass (96%), predominantly localized on the right side (87%) [5], as exemplified in the case under discussion. This phenomenon is purportedly attributed to the prolonged retention of endometrial cells on the right side due to gravitational forces, with potential protection of the left round ligament by the sigmoid colon. The inguinal mass may induce discomfort and exhibit an increase in size during menstruation, as reported by the patient.

The diagnostic process faced challenges preoperatively due to the presence of atypical radiographic characteristics and inconclusive FNA results. In traditional cases of endometriosis, ultrasonography and MRI serve as the primary modalities for diagnostic imaging. FNA biopsy has demonstrated notable accuracy in differentiating endometriosis from neoplastic or inflammatory conditions [8]. However, in the present scenario, FNA failed to yield a definitive diagnosis, prompting the pursuit of MRI for lesion characterization and localization. MRI showcases superior sensitivity and specificity compared to alternative imaging techniques in the identification of pelvic endometriosis. Pelvic MRI stands out as an exceptional tool for detecting the hemorrhagic components typical of endometriomas, with its broad imaging field and multiplanar capabilities facilitating precise mapping of multiple deeply infiltrating endometrial implants, thereby aiding in surgical strategizing [9]. Lesions typically exhibit low to intermediate signal intensity on T1-weighted MRI images, appear hypointense on T2-weighted images, and show minimal enhancement post administration of gadolinium-based contrast material. Additionally, hyperintense areas may be discerned on T1-weighted images, indicative of ectatic endometrial glands containing hemorrhagic material [10].

This case exemplifies the difficulties encountered when diagnosing uncommon manifestations of well-known illnesses. The presence of a detectable mass in the subcutaneous layers of the pelvic region is a nonspecific discovery. Nevertheless, in females vulnerable to endometriosis, we argue that the conventional belief of atypical expressions of a common disorder must be contemplated in the diagnostic process, as demonstrated by the exceptional imaging results in our patient with extrapelvic endometriosis in the canal of Nuck [11]. Initially, the disease was situated in an exceedingly rare location. Subsequently, the FNA yielded inconclusive results. Lastly, the imaging patterns identified through MRI in our patient were indicative of the condition. A palpable mass in the subcutaneous tissues of the pelvis is a nonspecific observation. However, in women at risk of endometriosis, we posit that the traditional notion of unique displays of a common ailment must be factored into the diagnostic framework, as exemplified by the extraordinary imaging findings in our patient with extrapelvic endometriosis in the canal of Nuck with elevated levels of CA 125 of 55 U/mL. The surgical removal of the affected tissue serves both diagnostic and therapeutic purposes for pelvic endometriosis. Strasser and Davis [12] have demonstrated that excision leads to a permanent alleviation of pain and acts as a curative measure. Instances of malignant transformation of endometriosis within the vulva and labia have been documented, with a reported transformation rate ranging from 0.7% to 1.0% [13]. Opting for a comprehensive surgical excision of the lesion is the preferred treatment approach and offers confirmation through histological analysis. Additionally, laparoscopy is warranted to assess potential concurrent pelvic and intra-abdominal endometriosis.

## Conclusions

We present a unique case of extraperitoneal endometriosis manifesting in the canal of Nuck. Endometriosis occurring in this location may manifest as a painless swelling in the groin and exhibit correlation with the menstrual cycle, possibly due to its anatomical distance from the uterus and lack of hormonal influence. Promt imaging techniques and surgical intervention are often necessary. Achieving an accurate diagnosis necessitates the application of both histological and immunocytochemical methodologies.
